# The Impact of Midline Deviation on Smile Attractiveness: A Cross-Sectional Study Among Laypersons

**DOI:** 10.7759/cureus.79479

**Published:** 2025-02-22

**Authors:** Lana Hodali, Nabil Massad

**Affiliations:** 1 Faculty of Dentistry, An-Najah National University, Nablus, PSE

**Keywords:** aesthetics, deviation, laypersons, midline, smile

## Abstract

Objective

This study aimed to assess laypersons' perceptions of smile aesthetics in relation to upper dental midline deviations and to determine whether adjacent facial structures influence their judgments.

Methodology

A cross-sectional study was conducted using two sets of photographs depicting midline deviations. The first set included six images showing only the lips, while the second set featured six images displaying the lips, chin, and two-thirds of the nose. Both sets presented deviations at increments of 0 mm (no shift), 1, 2, 3, 4, and 5 mm relative to the facial midline. The photographs were randomly labeled (A-F) and presented in a nonsequential order to prevent bias. Laypersons rated the attractiveness of each smile on a scale of 1 (very unattractive) to 10 (very attractive). Data were tabulated and analyzed using SPSS software version 22 (IBM Corp., Armonk, NY).

Results

Responses from 242 laypersons (127 females and 115 males) revealed that midline deviations of up to 3 mm were considered aesthetically acceptable across both photo sets. Friedman’s test indicated statistically significant differences in ratings for each midline shift in both groups (*P* < 0.001). However, no significant differences were observed between ratings for smiles showing only the lips and those including the lips, chin, and nose. Additionally, gender and age did not significantly influence perceptions of smile attractiveness.

Conclusions

Laypersons perceive midline deviations of up to 3 mm as attractive, regardless of whether adjacent facial structures are included in the images. These findings suggest that the presence of additional facial features does not significantly alter aesthetic judgments of midline deviations.

## Introduction

A smile plays a crucial role in personal attractiveness and significantly influences social interactions [1]. Beyond the shape, color, and alignment of teeth, smile aesthetics are also determined by the spatial dynamics of the smile. For instance, the buccal corridor can greatly affect the perceived fullness and harmony of the smile. Similarly, a gummy smile, characterized by an excessive display of gingiva, may disrupt the balance between teeth and soft tissue, potentially detracting from overall facial aesthetics [2, 3].

The dental midline, a key component of smile aesthetics, is typically defined by the alignment of the gingival papilla tip between the maxillary central incisors with the center of the philtrum on the upper lip. Deviations between the upper dental midline and the facial midline measured in millimeters from a perpendicular line at the glabella across the interpupillary distance can significantly affect dentofacial aesthetics [4, 5]. Research has explored the impact of dental-to-facial midline discrepancies on perceived attractiveness. Studies by Johnston et al. [6] and Beyer and Lindauer [4] found that discrepancies exceeding 2 mm were considered aesthetically unacceptable. In contrast, Shyagali et al. [7] reported that while midline deviations of up to 4 mm might go unnoticed, changes in the angulation of anterior teeth were perceived as unattractive.

Laypersons' aesthetic expectations are critical for achieving successful outcomes in orthodontic, restorative, and prosthetic dentistry. However, perceptions of an ideal smile, particularly regarding midline alignment, vary significantly among orthodontists, prosthodontists, and laypersons [7,8]. This variability poses challenges for practitioners striving to meet patient expectations, especially when aesthetics is a primary concern. Consequently, understanding the acceptable thresholds for midline deviation among laypersons is essential for delivering satisfactory aesthetic results.

Numerous studies have explored the threshold at which midline deviations become noticeable and impact smile aesthetics. However, findings reveal considerable variability in tolerance levels across different population groups, reflecting the influence of cultural, social, and individual factors on aesthetic preferences [7,9-11].

This variability highlights the need for further research, particularly in diverse cultural contexts. To date, no studies have examined these perceptions within the Palestinian population. This study aims to assess how midline deviations influence the aesthetic perceptions of smiles among Palestinian laypersons in the city of Nablus, as well as the role of adjacent anatomical structures in their aesthetic judgments.

## Materials and methods

This study aims to assess the influence of upper dental midline deviation on smile attractiveness as perceived by laypersons. Specifically, the objectives are to evaluate how different degrees of midline deviation (0-5 mm) affect aesthetic perception, determine whether the presence of additional facial features (chin and nose) influences attractiveness ratings compared to lip-only images, and analyze potential differences in perception based on demographic factors such as gender and age.

The study focused on laypersons in a specific area of Nablus, Palestine, which has an estimated population of 174,000 [12]. The required sample size was calculated using a 95% confidence level, a 5% margin of error, and an assumption of maximum variability (*P* = 0.5). Based on these parameters, the calculation determined that a minimum of 385 participants was necessary to ensure statistical representativeness. To enhance clarity and accessibility, the questionnaire was translated into Arabic. It included an introductory section detailing the study's background, objectives, voluntary nature of participation, and assurances of confidentiality and anonymity.

A pilot study involving 20 participants from the target population was conducted to evaluate the questionnaire's clarity, cultural relevance, and comprehensiveness. Feedback from the pilot study was used to refine the questions, ensuring they were easily understood and aligned with the study's objectives.

Inclusion criteria required participants to be laypersons with no formal dental training or prior orthodontic treatment, ensuring that their responses reflected pure aesthetic perceptions uninfluenced by professional knowledge or personal treatment history. Participants who did not complete the questionnaire were excluded, as incomplete responses could compromise data integrity.

Data collection was conducted through face-to-face interviews or via an electronic questionnaire accessed through QR codes or invitations. The questionnaire was divided into two sections. The first section collected demographic information, including the participant's gender and age. The second section asked participants to rate the attractiveness of smiles on a scale from 1 to 10, where 1 represented a very unattractive smile and 10 represented a very attractive one.

**Figure 1:**
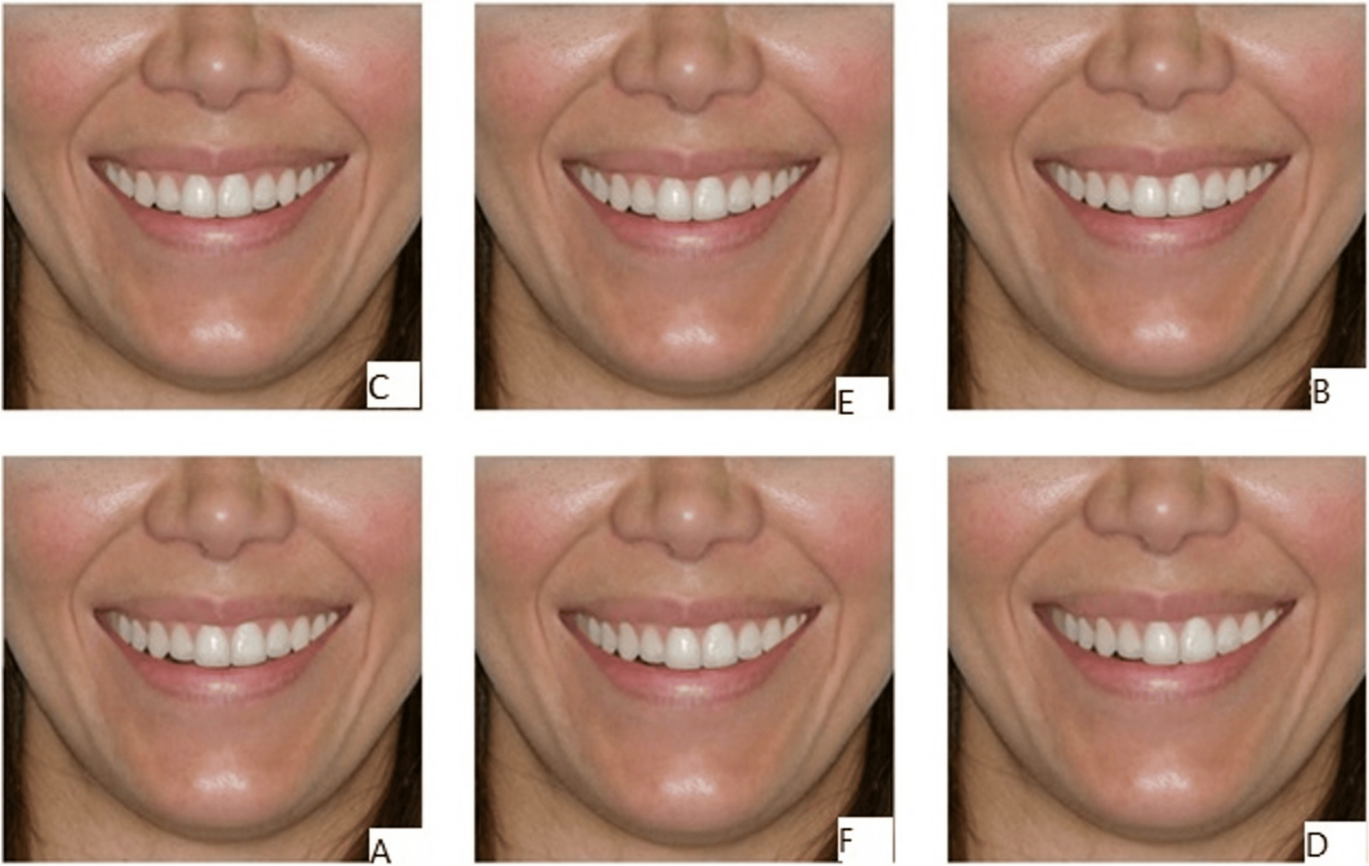
Randomly arranged photographs showing smiles, including the lips, chin, and two-thirds of the nose. Each of the photographs had a different midline deviation. Reprinted from Ferreira et al. [13], with permission from DentalPress Publishing.

**Figure 2:**
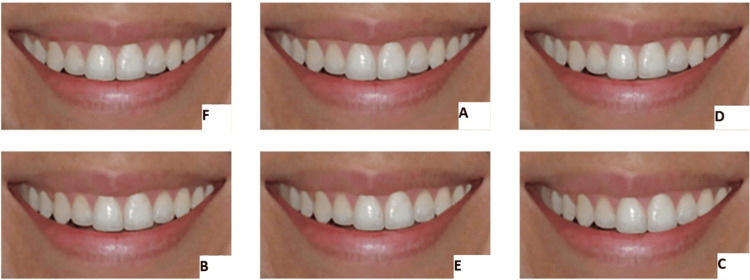
Randomly arranged photographs showing smiles, including the lips only. Each of the photographs had a different midline deviation. Reprinted from Ferreira et al [13], with permission from DentalPress Publishing.

The study utilized twelve photographs depicting varying degrees of upper dental midline deviation. The first six images showed the lips, chin, and part of the nose (Figure 1), while the remaining six displayed only the lips (Figure 2). Both sets (A and B) included deviations at increments of 0 mm (no shift), 1, 2, 3, 4, and 5 mm relative to the facial midline. To prevent bias, the photographs were labeled randomly (A to F) and presented in a non-sequential order. The images were sourced from a previous study [13], with permission obtained from the corresponding journal's editorial office for their reprinting. Ethical approval for the study was granted by the Institutional Review Committee (IRB) at An-Najah National University, Nablus, Palestine.

Data were organized in a spreadsheet and analyzed using IBM SPSS Statistics V. 22 (IBM Corp., Armonk, NY). SPSS calculated percentages of individual variables, and data normality was evaluated using the Kolmogorov-Smirnov and Shapiro-Wilk tests. We applied Friedman’s test, followed by the Wilcoxon test for multiple pairwise comparisons, to assess the effect of midline deviation on the perception of smile aesthetics. The Mann-Whitney test was used to compare unpaired variables. Pearson's correlation coefficient assessed the association between ratings from photographs showing the entire face and those showing only the lips.

## Results

The study analyzed responses from 242 laypersons, comprising 52% females and 48% males, with participants distributed across various age groups, as outlined in Table 1. The analysis showed no statistically significant differences in smile ratings between genders, as indicated by a Mann-Whitney U test result with a *P*-value of 0.776. Mean ratings for smiles with or without midline deviations were comparable between male and female participants across all categories, with minor variations that lacked statistical significance. These results suggest that gender does not influence the perception of smile aesthetics, reinforcing that midline deviations up to 3 mm are generally perceived as acceptable by all participants, irrespective of gender. Similarly, the analysis revealed no statistically significant differences in smile ratings across age groups, with *P*-values exceeding 0.05 for all pairwise comparisons. Mean ratings for midline deviations up to 3 mm were consistent among all age groups, indicating a shared perception of aesthetic acceptability. Despite minor variations, these differences were not statistically meaningful, suggesting that age does not influence the evaluation of smile aesthetics.

**Table 1:** The gender distribution of participants across various age groups.

		20-25 years	26-31 years	32-37 years	38-43 years	44-49 years
Female, *n* (%)	127 (52%)	36 (28%)	34 (27%)	29 (23%)	17 (13%)	11 (9%)
Male, *n* (%)	115 (48%)	36 (31%)	33 (29%)	29 (25%)	11 (9%)	6 (5%)

Results indicated that smiles with no midline deviation or slight deviations of 1-3 mm were generally rated attractive. Specifically, 28.95% of participants rated a smile with no deviation highly (score of 8). As the midline deviation increased, ratings for attractiveness declined, particularly for 4 and 5 mm deviations, which received more mixed or lower scores. Statistical analysis further supported these findings. Friedman's test revealed significant differences in attractiveness ratings across deviations (*P* < 0.001), and the Wilcoxon test confirmed that deviations up to 3 mm were rated significantly higher than larger deviations (Table 2).

**Table 2:** Percentage score given by laypersons.

Scores	Smile including the face (%)						Smile including only the lips (%)					
	A (3 mm)	B (2 mm)	C (0 mm)	D (5 mm)	E (1 mm)	F (4 mm)	A (1 mm)	B (3 mm)	C (5 mm)	D (2 mm)	E (4 mm)	F (0 mm)
1	1.75	0.88	0.88	16.67	1.75	10.53	6.14	7.02	18.42	7.02	18.42	6.14
2	4.39	2.63	0.88	7.02	2.63	6.14	3.51	7.89	11.40	1.75	7.02	3.51
3	5.26	7.02	2.63	9.65	4.39	12.28	6.14	7.02	8.77	2.63	10.53	7.02
4	7.89	8.77	7.89	10.53	7.89	11.40	7.89	7.02	8.77	5.26	9.65	3.51
5	18.42	14.91	8.77	13.16	14.04	12.28	8.77	13.16	14.91	5.26	9.65	11.40
6	11.40	16.67	7.89	9.65	9.65	8.77	8.77	7.89	5.26	7.02	10.53	11.40
7	14.91	14.04	11.40	13.16	12.28	12.28	9.65	12.28	14.04	16.67	4.39	9.65
8	20.18	21.05	28.95	12.28	21.05	12.28	22.81	23.68	8.77	20.18	17.54	17.54
9	9.65	9.65	19.30	6.14	18.42	9.65	19.30	8.77	4.39	20.18	7.02	18.42
10	6.14	4.39	11.40	1.75	7.89	4.39	7.02	5.26	5.26	14.04	5.26	11.40

The Mann-Whitney test showed no statistically significant difference in median attractiveness ratings between the two photograph types (*P* > 0.05), suggesting that the inclusion of adjacent facial features did not influence layperson perceptions of smile aesthetics (Table 3).

**Table 3:** Mann-Whitney test for attractiveness ratings between photograph types.

Photograph type	Median score	Interquartile range (IQR)	Mann-Whitney U test	*P*-value
Lips, chin, and nose	7	3	25,992	>0.05
Lips only	7	4		

Additionally, a weak positive correlation (*r* = 0.224, *P* < 0.001) was observed between ratings for the two photograph types, indicating some consistency in laypersons' ratings across both formats (Table 4).

**Table 4:** Pearson’s correlation coefficient between photograph types.

Comparisons	Pearson’s correlation coefficient (r)	*P*-value
Lips, chin, and nose vs. lips only	0.224	<0.001

## Discussion

Understanding the thresholds at which midline deviations become noticeable to patients is critical in orthodontic and restorative treatments, as these deviations significantly influence perceived aesthetics. Studies report varying thresholds for acceptable midline deviations across different populations. For example, Ker et al. [14] found an acceptable threshold ranging from 2.9 to 4.3 mm, while Tahir et al. [15] and Pinho et al. [8] observed that laypersons often failed to detect midline shifts of up to 4 mm. Similarly, Shyagali et al. [7] noted that deviations of 2 mm or more are frequently perceptible, suggesting that even minor discrepancies can disrupt smile harmony. The present study aligns with these findings, demonstrating that laypersons generally accept smiles with midline deviations of up to 3 mm, regardless of whether the full lower face or only the lips are visible.

Cultural and demographic factors play a significant role in shaping acceptable thresholds for midline deviations. McLeod et al. [16] found that Canadian laypersons were more discerning, accepting deviations of up to 1.83 mm, compared to 2.9 mm among Americans. Musa et al. [17] emphasized that perceptions vary by ethnicity, profession, and socio-demographic characteristics. Zhang et al. [9] reported a lower tolerance for deviations exceeding 2 mm in men with tapered facial shapes. Similarly, Williams et al. [18] demonstrated that facial type and sex influence perception, with broader-faced (euryprosopic) individuals and males showing greater tolerance for minor deviations.

This study found no significant difference in midline deviation scoring between images showing only the lips and those including additional facial features. This is consistent with Springer et al. [3], who reported similar aesthetic ratings between full-face and lower-face perspectives, suggesting that viewing angle has minimal impact on midline deviation perception. However, Ferreira et al. [13] found that deviations as small as 1 mm were detectable when adjacent facial structures were visible, compared to 2 mm when only the lips were shown, indicating that context can influence visibility. Musa et al. [17] also highlighted that smile-associated structures affect midline perception, with full-face views potentially reducing the focus on specific smile details. Flores-Mir et al. [19] further noted that dental aesthetics have less impact in full-face views compared to close-up or lower-face perspectives, as subtle dental changes are less noticeable within a broader facial context.

Although previous studies have examined midline deviation thresholds in various populations, none have been conducted in Palestine. Our study offers unique contributions by focusing on lay perceptions within a Palestinian cohort, thereby addressing a significant gap in understanding the cultural influences on aesthetic judgment. Moreover, by comparing images showing only the lips with those displaying the full lower face, we shed new light on how facial context affects the perception of midline deviations. By integrating diverse cultural and sociodemographic factors, our research not only enriches global understanding but also provides valuable, context-specific insights for orthodontic and restorative practices in Palestine.

This study has limitations that should be addressed in future research to enhance our understanding of lay perceptions across diverse populations, including the broader Palestinian community. The relatively small sample size, drawn exclusively from Nablus - a diverse urban center - may not fully capture the range of perceptions among laypersons from different socio-demographic and regional backgrounds. Consequently, caution should be exercised when generalizing these findings to all Palestinians, as regional variations within Palestine may exist. Future studies should include participants from multiple regions to validate and extend our observations. Additionally, the use of predetermined photographs from prior studies may not account for the full variability in smile aesthetics and midline deviations. The cross-sectional design limits our ability to infer causality, and reliance on self-reported evaluations may introduce response bias. Future studies should aim to include larger, more regionally diverse samples and utilize alternative methodologies to yield more generalizable insights. Where feasible, increasing the sample size would enhance precision and statistical power, thereby strengthening the validity and reliability of the findings.

## Conclusions

Laypersons perceive midline deviations of up to 3 mm as acceptable and aesthetically pleasing, regardless of whether adjacent facial structures are visible. The study found no significant influence of demographic factors such as gender and age on these perceptions, underscoring the shared aesthetic standards within the study population. These findings provide valuable insights for orthodontic and restorative dental treatment planning, suggesting that small midline deviations may not compromise smile aesthetics from the layperson's perspective. Future research with broader population samples could help refine our understanding of aesthetic thresholds and cultural influences on smile perception.
